# Clinical Impact of PET With ^18^F-FDG and ^11^C-PIB in Patients With Dementia in a Developing Country

**DOI:** 10.3389/fneur.2021.630958

**Published:** 2021-05-04

**Authors:** Andres Damian, Fabiola Portugal, Nicolas Niell, Adriana Quagliata, Karina Bayardo, Omar Alonso, Rodolfo Ferrando

**Affiliations:** ^1^Centro Uruguayo de Imagenología Molecular (CUDIM), Montevideo, Uruguay; ^2^Centro de Medicina Nuclear e Imagenología Molecular, Hospital de Clínicas, Universidad de la República (UdelaR), Montevideo, Uruguay

**Keywords:** PET, Alzheimer's, amyloid, dementia, neuroimaging, biomarkers, clinical diagnosis, impact

## Abstract

**Introduction:** The objective of this study was to evaluate the clinical impact PET with ^18^F-FDG and ^11^C-PIB in patients with dementia in a developing country.

**Methodology:** Retrospective study of the patients referred for the evaluation of dementia to the only PET center in Uruguay. A total of 248 patients were identified, from which 70 patients were included based on the availability of medical history and clinical follow-up. Main outcomes included change in diagnosis, diagnostic dilemma and AD treatment. We evaluated the association of clinical outcomes with PET concordance with baseline diagnosis, diagnostic dilemma, level of education, AD pathology/Non-AD pathology (AD/Non-AD), baseline diagnosis and ^11^C-PIB PET result.

**Results:** Baseline clinical diagnosis was concordant with ^18^F-FDG and ^11^C-PIB PET results in 64.7 and 77.1% of the patients, respectively. Change in diagnosis after PET was identified in 30.0% of the patients and was associated with discordant ^18^F-FDG (*p* = 0.002) and ^11^C-PIB (*p* < 0.001) PET results, previous diagnostic dilemma (*p* = 0.005), low education (*p* = 0.027), Non-AD baseline diagnosis (*p* = 0.027), and negative ^11^C-PIB PET result (*p* < 0.001). Only the last variable remained significant in the multivariate analysis (adjusted *p* = 0.038). Diagnostic dilemma decreased after PET from 15.7 to 7.1% (*p* = 0.11) and was associated with Non-AD diagnosis (*p* = 0.002) and negative ^11^C-PIB PET result (*p* = 0.003). Change in AD treatment after PET occurred in 45.7% of the patients.

**Conclusion:** ^18^F-FDG and ^11^C-PIB PET had a significant clinical impact in terms of change in diagnosis and treatment in patients with dementia in a developing country, similar to that reported in high-income countries.

## Introduction

The expected increase in the prevalence of neurodegenerative diseases in the coming years will particularly affect low- and middle-income countries (1). In this context, it is imperative to evaluate the clinical impact of dementia biomarkers to gather relevant information for the construction of rational diagnostic algorithms.

There is a considerable amount of evidence supporting the clinical use of ^18^F-FDG PET in the evaluation of patients with cognitive impairment (2, 3). This information has led to the incorporation of ^18^F-FDG into clinical and research guidelines in dementia (4, 5). In the last 15 years, PET with amyloid tracers has also gained ground in the field, becoming one of the leading amyloid biomarkers (5, 6).

Access to high-cost diagnostic biomarkers, such as PET studies, shows significant global heterogeneity, with clear inequities between high- and low- and middle-income countries. PET cameras per million inhabitants can vary from 0.007 to 3.2 between low- and middle- and high-income countries, respectively (7). In this context, the Latin America and Caribbean region (LAC) averages 0.47 PET cameras/million inhabitants, a number clearly below the recommended 2–2.5 (7, 8). The low accessibility to high-cost biomarkers is generally accentuated in populations of public health systems and outside of large cities, which has determined their low representation in the scientific literature (9, 10).

Recently, important clinical studies are been carried out to stablish the clinical impact of amyloid and ^18^F-FDG PET in the clinical practice (11–15). Although there is now significant evidence about the clinical impact of incorporating these tools in the assessment of patients with cognitive impairment, most of the literature comes from high-income countries. There is little evidence on the clinical impact of these tools in less developed health systems and populations that are usually underrepresented in the clinical literature in the field of dementia.

The objective of the present work is to study the clinical impact of PET with ^18^F-FDG and ^11^C-PIB in patients with cognitive impairment referred for clinical evaluation in a developing country.

## Methodology

### Study Population

We retrospectively reviewed the Uruguayan Center of Molecular Imaging (CUDIM) database, identifying patients who had undergone both ^18^F-FDG and ^11^C-PIB PET/CT between April 2011 and May 2020. All patients had been referred for evaluation of cognitive impairment from different public and private specialized medical centers because of an uncertain diagnosis despite a complete clinical evaluation by a neurologist, neuropsychological assessment and structural imaging. Of the 248 available patients, we had access to the medical history and clinical follow-up (mean follow-up 4.5 years, range 0.5–8 years) in 70 cases, which were finally included in the analysis. All 70 patients were evaluated with ^11^C-PIB PET/CT and 51 of them also underwent ^18^F-FDG PET/CT. A summary of patient characteristics is presented in Table 1.

**Table 1:** Population characteristics.

**All patients (*n =* 248)**			
Age (mean ± SD; range)	70.2	±9.62	50–87
Gender (% female)	142	57.3%	
**Patients with follow-up (*n =* 70)**			
Age (mean ± SD; range)	67.29	±10.22	48–86
Gender (% female)	42	60%	
Formal education (mean ± SD; range)	11.69	±5.5	0–23
MMSE (mean ± SD; range)	23.84	5.66	5–30
Disease duration in years (mean ± SD; range)	3.04	3.10	0.5–20
**Baseline diagnosis (n, %)**			
AD	61	87.1%	
Non-AD	9	12.8%	

*AD, Alzheimer's Disease; MMSE, Mini-Mental State Examination; SD, Standard Deviation*.

### Clinical Evaluation

A complete medical history from the patient and a close informant as well as a detailed general and neurological physical examination was performed by a dementia specialist in all patients. Laboratory tests included complete blood cell count, calcium, glucose, renal and liver function, vitamin B12, folate, thyroid stimulating hormone and serological tests for syphilis and HIV. The global cognitive function was assessed with the Mini-Mental State Examination (MMSE) and the Addenbrooke's Cognitive Examination (ACE). The neuropsychological evaluation consisted of tests evaluating memory, language, praxis, visual-spatial abilities, attention and executive function. Test performed in all centers included the Rey Auditory Verbal Learning test, category fluency test, Boston Naming test, Rey-Osterrieth Complex Figure test, clock drawing test, forward and backward digit span tests, Trail Making tests A and B, Stroop Color Test, Symbol Digit Modalities test and the Neuropsychiatric Inventory scale. Dementia severity was assessed with the Clinical Dementia Rating scale (CDR). All patients underwent structural magnetic resonance imaging (MRI) of the brain.

### Image Acquisition and Interpretation

PET/CT imaging was performed within 1 month from referral. Both ^18^F-FDG and ^11^C-PIB images were obtained on a GE Discovery 690 or a GE Discovery STE PET/CT scanner on separate days within a 2-month period. For ^18^F-FDG PET, patients fasted for 6 h and abstained from tea, coffee, alcohol and nicotine. Images were performed if blood glucose levels were below 150 mg/dl. Patients received an intravenous injection of 3.0 MBq/kg of ^18^F-FDG in a dimmed quiet room with no external stimuli. Forty minutes later, 3D PET/CT images were acquired. For ^11^C-PIB PET/CT, the patient was positioned in the scanner, a low dose CT was acquired for attenuation correction and anatomical correlation, and a full dynamic 3D PET/CT acquisition was performed after the intravenous administration of 4.0 MBq/kg of the radiotracer.

Images were analyzed and interpreted by at least two experienced nuclear medicine physicians independently and the discrepancies were solved by consensus. ^18^F-FDG PET results were reported following previously described criteria (2). Briefly, an Alzheimer's disease (AD) pattern was reported when hypometabolism in parietotemporal cortex and posterior cingulate gyrus was detected and metabolism was preserved in occipital and sensory-motor cortex, basal ganglia and cerebellum. Other characteristic patterns of neurodegenerative dementia were also considered, including frontal and temporal hypometabolism in frontotemporal dementia (FTD) and posterior parietal and occipital hypometabolism in Lewy body dementia (LBD) (2). If no such pattern was present on ^18^F-FDG PET images, the study was reported as a non-degenerative disease. Quantification through Z-score maps was available for interpretation of all ^18^F-FDG PET images (CortexID, GE Heathcare, UK). ^11^C-PIB PET was reported as positive or negative considering the presence or absence of significant cortical uptake, as described elsewhere (6, 16).

### Study Approval and Patient Consent

Written informed consent was obtained from all patients or caregivers. The study was approved by the ethics committee of the Uruguayan Center of Molecular Imaging.

### Data Analysis

Based on the methodology of previous reports with similar approaches (17, 18), baseline clinical diagnosis before PET was classified as associated with Alzheimer's disease pathology (AD) when the patient had a diagnosis of possible or probable AD based on the NIA-AA criteria (*n* = 38), amnestic mild cognitive impairment (MCI, *n* = 22) or LBD (*n* = 1). Non-AD baseline diagnosis was considered when the patient had a previous diagnosis of FTD (*n* = 5), semantic dementia (*n* = 1) or non-fluent primary progressive aphasia (*n* = 3). Baseline diagnosis on referral was based on previous clinical, neuropsychological and structural imaging information. The patients that have been referred with more than one clinical diagnosis were classified as diagnostic dilemmas and the first diagnosis listed was considered for AD/Non-AD classification. Concordance between PET and baseline diagnosis was established considering ^18^F-FDG and ^11^C-PIB patterns described above. LBD was considered within the AD category because of the high prevalence of amyloid deposits in the disease. After PET/CT imaging, the reports were disclosed to the referring physician and incorporated in the regular diagnostic work-up of the patients. Changes in diagnosis (whether or not the diagnosis change after disclosure of PET result), pharmacological AD treatment (addition or suspension of AD related treatment including donepezil, memantine, galantamine, or rivastigmine) and diagnostic dilemma (whether or not the patient had more than one clinical diagnosis) were evaluated by three experienced physicians and considered as the outcomes for the study. The definite diagnosis was the main diagnosis defined by the neurologist after the disclosure of PET results, considering clinical follow-up and all neuropsychological, laboratory and imaging information. For the statistical analysis, the association of the outcomes with the following variables was assessed individually using Fisher's exact test: PET concordance with baseline diagnosis, formal education (≤ 9 years or > 9 years), AD/Non-AD baseline diagnosis, baseline diagnostic dilemma and ^11^C-PIB PET result. Additionally, logistic regression analysis was performed exploring the following predictors of the outcomes: baseline AD/Non-AD diagnosis, baseline diagnostic dilemma, discordance of ^11^C-PIB PET with baseline diagnosis, discordance of ^18^F-FDG PET with baseline diagnosis and ^11^C-PIB PET result. A *p* value lower than 0.05 was considered significant.

## Results

### Concordance Between PET Studies and Baseline Diagnosis

The concordance between PET results and previous clinical diagnosis was 77.1% for ^11^C-PIB PET and 64.7% for ^18^F-FDG PET. No significant differences were found between ^18^F-FDG and ^11^C-PIB PET (p = 0.23). Considering only the MCI subgroup, we found a 77.3 and 70.5% concordance with previous diagnosis for ^11^C-PIB and ^18^F-FDG, respectively, with no significant differences in comparison with the rest of the patients (*p* = 0.98 for ^11^C-PIB and *p* = 0.77 for ^18^F-FDG). ^11^C-PIB and ^18^F-FDG PET agreed in the classification of 90.2% of the patients.

### Change in Diagnosis After PET

**Figure 1:**
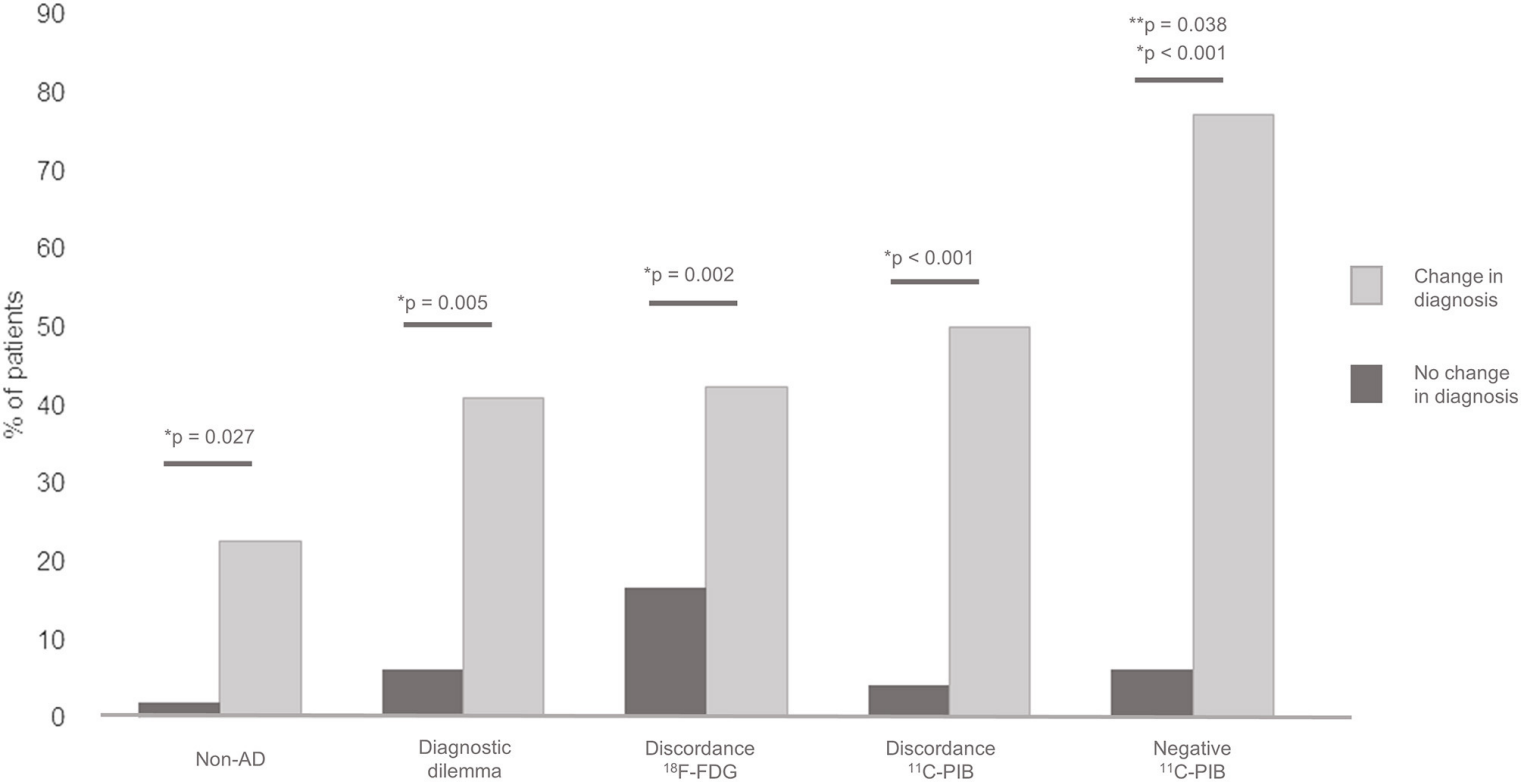
Association between diagnostic change and baseline Non-AD diagnosis, previous diagnostic dilemma, ^18^F-FDG PET discordance with baseline diagnosis, ^11^C-PIB PET discordance with baseline diagnosis and negative ^11^C-PIB PET result. *Individual comparison significance. **Mutivariate logistic regression significance.

**Figure 2:**
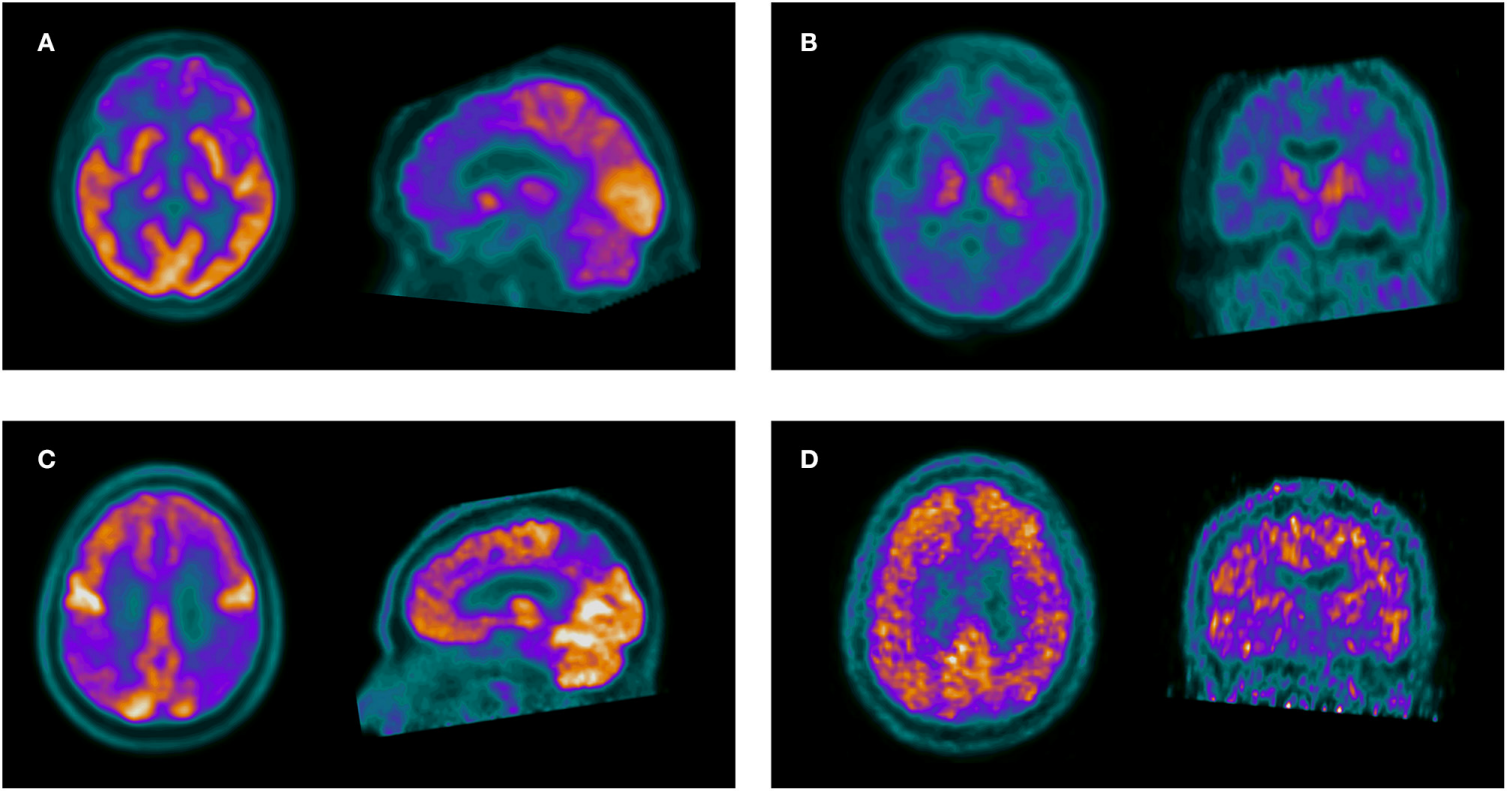
The upper row shows a 56-year-old woman with memory complaints and change in behavior, referred with overlapping symptoms of AD, FTD and a baseline diagnosis of AD. Axial and sagittal slices of 18F-FDG PET **(A)** show frontal hypometabolism suggestive of FTD. Axial and coronal ^11^C-PIB PET slices **(B)** are negative for amyloid deposition, confirming FTD. The lower row shows a 68-year-old male with symptoms of a non-fluent progressive aphasia. ^18^F-FDG PET **(C)** shows posterior parietal and precuneus hypometabolism suggestive of AD. ^11^C-PIB PET **(D)** confirms AD pathology.

Overall change in diagnosis after PET was observed in 30.0% of the patients. When compared separately, a significant association was found between the discordance of PET with baseline diagnosis and the change in diagnosis after PET (*p* < 0.001 for ^11^C-PIB and *p* = 0.002 for ^18^F-FDG PET). In addition, the change in diagnosis after PET was associated with lower educational level (*p* = 0.027), Non-AD baseline classification (*p* = 0.027), the presence of a diagnostic dilemma prior to PET (*p* 0.005) and a negative ^11^C-PIB PET result (*p* < 0.001). The MCI subgroup showed a 18.2% in change in diagnosis, with no significant differences in comparison with the rest of the patients (*p* = 0.17). In the multiple logistic regression model, only the negative result of the ^11^C-PIB PET study remained statistically significant (β-coefficient = −2.43, Standard Error = 1.09, *p* = 0.038) Figure 1. Change in diagnosis was observed in overall in 21 patients, 14 with baseline AD classification (9 with AD, 4 with amnestic MCI and 1 with LBD) and 7 with baseline non-AD classification (5 with FTD, 1 with SD and 1 with non-fluent APP). The patients in which a change in diagnosis after PET was observed had a disease duration of 3.5 ± 2.6 years. No diagnostic changes were found in patients with both ^18^F-FDG and ^11^C-PIB PET results concordant with baseline diagnosis. Figure 2 shows two examples of patients in which PET results determined a change in diagnosis.

### Diagnostic Dilemma

The diagnostic dilemma decreased from 15.7 to 7.1% after PET, even though the decrease was not statistically significant (*p* = 0.11). Nevertheless, the change in dilemma was associated with baseline Non-AD classification (*p* = 0.002), and negative ^11^C-PIB PET result (*p* = 0.03). In the logistic regression analysis, no significant results were obtained for this outcome.

### Change in Treatment

A change in pharmacological treatment related to AD after PET was observed in 45.7% of the patients, either including or retiring AD related pharmaceuticals. There was no significant association between treatment change and ^18^F-FDG or ^11^C-PIB PET discordance, baseline AD/Non-AD-diagnosis, baseline diagnostic dilemma or ^11^C-PIB PET result.

## Discussion

In the present study, the clinical impact of ^18^F-FDG and ^11^C-PIB PET/CT was assessed in patients with cognitive impairment, exploring the change in diagnosis, specific AD treatment and diagnostic dilemma after PET. The study aimed to provide evidence about the usefulness of these techniques focusing on a particular cohort of patients referred from public and private institutions in a developing country like Uruguay. This constitutes the main strength of the study, given that populations from less developed health systems tend to be underrepresented in clinical studies with high-cost techniques. It is worth to notice that the study involved patients from both the public and private systems and represents the nationwide experience, since the institution where the study was carried out is the only PET center in Uruguay, providing assistance to the whole population.

Firstly, we observed a high overall agreement between PET results and baseline clinical diagnosis (70% of the patients). Although this phenomenon may vary depending on the characteristics of the population studied and the previous clinical and neuropsychological characterization, a high concordance has been reported, associated with a confirmatory role of PET studies in a significant proportion of the patients (17, 18).

The agreement was higher in the MCI subgroup, with 77.3 and 70.7% concordance for ^11^C-PIB and ^18^F-FDG, respectively. The percentage of concordance previously reported for MCI patients has been variable. Lage et al. showed a concordance of 57 and 20% for ^11^C-PIB and ^18^F-FDG (17), while Sánchez-Juan et al. described a concordance of 80% for both radiotracers (18). In our region, Chrem Mendez et al. described a concordance of 68.8% for ^11^C-PIB (19) and Coutinho et al. showed 37% positive ^11^C-PIB results in amnestic MCI patients (20).

Regarding the clinical contribution of PET studies, we observed a change in diagnosis in 30% of the patients. Previous studies have shown variable results. In a recent systematic review by Fantoni et al. (13) amyloid PET resulted in a revised diagnosis in 31% of the cases. Other studies that incorporate both ^18^F-FDG and ^11^C-PIB PET reported changes of 9% (18) and 17% (17). These differences may vary depending on the methodological design and the clinical setting in which the study is performed, with reported values that can reach up to 79% (21–31). In the MCI subgroup we found a change in diagnosis in 18.2% of the patients, less than in the complete group but not statistically different. Nevertheless, it should be considered that all these patients had amnestic MCI and the impact of PET in terms of change in diagnosis may be higher in non-amnestic MCI patients. In concordance with previous results from other groups, the change in diagnosis after PET in our series was associated with the presence of a previous diagnostic dilemma, which highlights the importance of PET imaging in patients with a challenging diagnosis.

Both ^11^C-PIB and ^18^F-FDG PET discordance with baseline diagnosis were associated with a diagnostic change, with ^11^C-PIB PET discrepancy as the most significant variable of the two. Similar results have been reported by Sánchez-Juan et al. (18) and Lage et al. (17) showing that amyloid PET discordance was the factor that most influenced the change in diagnosis. Moreover, ^11^C-PIB PET result was the only variable that remained significant in the multivariate analysis. These results remark the importance of the evaluation of amyloid deposits in the brain for the referring physician. Several studies have demonstrated the high negative predictive value of amyloid PET, with reported values of up to 100% (32, 33). Thus, a negative result practically rules out AD, providing critical information for the physician to change the diagnosis.

It should be noted that although our study was carried out in a different population than most of the previous reports, the results were similar anyway, highlighting the importance of amyloid imaging as a determining factor for clinical decisions. A particularly interesting finding was the association between a low level of formal education and the change in diagnosis after PET imaging. Previous studies have described that dementia diagnosis may be challenging in individuals with low literacy. Low education can affect the formal testing of cognitive performance and motor skills (34–36). It is therefore likely that these patients might benefit more from the inclusion of biomarkers in their diagnostic workup, because of difficulties that may arise in clinical and neuropsychological assessment due to the low education level (37, 38). Change in diagnosis was also associated with baseline non-AD classification, but this result should be taken carefully since the vast majority of our patients had a previous AD diagnosis. When evaluating the change in diagnostic dilemma after PET, an association with Non-AD diagnosis was also found.

We observed a 45.7% change in AD treatment after PET. Other authors have reported similar values, with associations with PIB discordance that were not significant in our analysis (17, 18). Recently, Rabinovici et al. reported results of the multicenter IDEAS study that included 13.444 patients evaluated with amyloid PET in the USA. They described a change in patient management in 60.2% of the patients with MCI and 63.5% of the patients with AD, mostly related with specific drug treatment, which changed in 43.6% of patients with MCI and 44.9% of patients with dementia (11).

Even though only nine countries in the LAC region have cyclotrons for the production of radiotracers, the access to PET in the region has improved in the last few years, with an annual growth of ~21% (7, 8). Access to amyloid tracers has also improved and the proposed A-T-N criteria are increasingly being applied to classify the patients in research studies (19, 20, 39–44). Regarding the clinical utility of amyloid PET, Chrem Mendez et al. described 76.2% concordance of ^11^C-PIB PET with baseline diagnosis in patients with AD, and a range of concordance of 54.5–100% in other forms of cognitive impairment (19). It is important to emphasize on the complementary role of ^18^F-FDG and ^11^C-PIB in the diagnosis of patients with dementia, since the patterns of hypometabolism on the ^18^F-FDG scan can help to distinguish between different clinical entities in amyloid negative patients. This issue has been recently addressed in our region by Coutinho et al. (20) and Parmera et al. (44). A single ^18^F-FDG PET scan can be enough to provide an accurate diagnosis when a disease-specific hypometabolic pattern is identified, avoiding the need for more expensive techniques like amyloid PET.

The main limitation of our study is the sample size, mostly affecting the subgroup of Non-AD patients. Also, our sample did not include patients with other forms of cognitive impairment at baseline diagnosis, like non-amnestic MCI, vascular dementia, atypical parkinsonism or Parkinson's dementia, in which ^18^F-FDG or amyloid PET have proved useful for clinical characterization (45, 46). Another limitation is the lack of availability of tau biomarkers that are currently under development in our center. Nevertheless, several publications from other authors included similar sample sizes and the literature from developing countries is still very limited. Racial/ethnic disparities can influence dementia risk and care. The inclusion of underrepresented populations in dementia science represents an urgent need for diverse perspectives to protect public health (47). This constitutes the main strength of our work.

The results presented provide important information about the clinical impact of PET studies in developing countries. New prospective studies including larger populations are needed to evaluate the efficacy of these techniques in this setting. Comparison with other biomarkers and cost-effectiveness analysis will be needed for the inclusion of these tools in the diagnostic algorithms of patients with dementia taking into account the optimization of available resources.

## Data Availability Statement

The raw data supporting the conclusions of this article will be made available by the authors, without undue reservation.

## Ethics Statement

The studies involving human participants were reviewed and approved by Comité de Ética del Centro Uruguayo de Imagenología Molecular. The patients/participants provided their written informed consent to participate in this study.

## Author Contributions

AD and RF conception and design of the research. AD, FP, AQ, KB, and NN data collection. AD, RF, and FP statistical analysis, creation of the tables, figures, and writing of the manuscript. AD, FP, AQ, NN, KB, OA, and RF reviewed the manuscript and approved the final version. All authors contributed to the article and approved the submitted version.

## Conflict of Interest

The authors declare that the research was conducted in the absence of any commercial or financial relationships that could be construed as a potential conflict of interest.

## References

[1] Custodio N Wheelock A Thumala D Slachevsky A. Dementia in Latin America: epidemiological evidence and implications for public policy. Front Aging Neurosci. (2017) 9:221. 10.3389/fnagi.2017.00221 28751861 PMC5508025

[2] Schöll M Damián A Engler H. Fluorodeoxyglucose PET in neurology and psychiatry. PET Clin. (2014) 9:371–90. 10.1016/j.cpet.2014.07.005 26050943

[3] Engler H Damian A Bentancourt C. PET and the multitracer concept in the study of neurodegenerative diseases. Dement Neuropsychol. (2015) 9:343–9. 10.1590/1980-57642015DN94000343 29213983 PMC5619316

[4] Jack CR Bennett DA Blennow K Carrillo MC Dunn B Haeberlein SB. NIA-AA research framework: toward a biological definition of Alzheimer's disease. Alzheimer's Dementia. (2018) 14:535–62. 10.1016/j.jalz.2018.02.018 29653606 PMC5958625

[5] McKhann GM Knopman DS Chertkow H Hyman BT Jack CR Kawas CH. The diagnosis of dementia due to Alzheimer's disease: recommendations from the national institute on aging-Alzheimer's association workgroups on diagnostic guidelines for Alzheimer's disease. Alzheimer's Dementia. (2011) 7:263–9. 10.1016/j.jalz.2011.03.005 21514250 PMC3312024

[6] Johnson KA Minoshima S Bohnen NI Donohoe KJ Foster NL Herscovitch P. Appropriate use criteria for amyloid PET: a report of the amyloid imaging task force, the society of nuclear medicine and molecular imaging, and the Alzheimer's association. Alzheimer's Dementia. (2013) 9:e-1-16. 10.1016/j.jalz.2013.01.002 23360977 PMC3733252

[7] Paez D Giammarile F Orellana P. Nuclear medicine: a global perspective. Clin Trans Imaging. (2020) 8:51–3. 10.1007/s40336-020-00359-z

[8] Páez D Orellana P Gutiérrez C Ramirez R Mut F Torres L. Current status of nuclear medicine practice in Latin America and the Caribbean. J Nucl Med. (2015) 56:1629–34. 10.2967/jnumed.114.148932 26229143

[9] Parra MA Baez S Sedeño L Gonzalez Campo C Santamaría-García H Aprahamian I. Dementia in Latin America: paving the way toward a regional action plan. Alzheimer's Dement. (2020) 17:295–313. 10.1002/alz.12202 33634602 PMC7984223

[10] Parra MA Baez S Allegri R Nitrini R Lopera F Slachevsky A. Dementia in Latin America assessing the present and envisioning the future. Neurology. (2018) 90:222–31. 10.1212/WNL.0000000000004897 29305437 PMC5791795

[11] Rabinovici GD Gatsonis C Apgar C Chaudhary K Gareen I Hanna L. Association of amyloid positron emission tomography with subsequent change in clinical management among medicare beneficiaries with mild cognitive impairment or dementia. JAMA - J Am Med Assoc. (2019) 321:1286–94. 10.1001/jama.2019.2000 PMC6450276 30938796

[12] Brendel M Schnabel J Schönecker S Wagner L Brendel E Meyer-Wilmes J. Additive value of amyloid-PET in routine cases of clinical dementia work-up after FDG-PET. Eur J Nucl Med Mol Imaging. (2017) 44:2239–48. 10.1007/s00259-017-3832-z 28932894

[13] Fantoni ER Chalkidou A O'Brien JT Farrar G Hammers A. A systematic review and aggregated analysis on the impact of amyloid PET Brain imaging on the diagnosis, diagnostic confidence, and management of patients being evaluated for Alzheimer's disease. J Alzheimer's Dis. (2018) 63:783–96. 10.3233/JAD-171093 29689725 PMC5929301

[14] Leuzy A Savitcheva I Chiotis K Lilja J Andersen P Bogdanovic N. Clinical impact of [18 F]flutemetamol PET among memory clinic patients with an unclear diagnosis. Eur J Nucl Med Mol Imaging. (2019) 46:1276–86. 10.1007/s00259-019-04297-5 30915522 PMC6486908

[15] Perini G Rodriguez-Vieitez E Kadir A Sala A Savitcheva I Nordberg A. Clinical impact of 18F-FDG-PET among memory clinic patients with uncertain diagnosis. Eur J Nucl Med Mol Imaging. (2020) 48:612–22. 10.1007/s00259-020-04969-7 32734458 PMC7835147

[16] Klunk WE Engler H Nordberg A Wang Y Blomqvist G Holt DP. Imaging brain amyloid in Alzheimer's disease with Pittsburgh compound-B. Ann Neurol. (2004) 55:306–19. 10.1002/ana.20009 14991808

[17] Lage C Suarez AG Pozueta A Riancho J Kazimierczak M Bravo M. Utility of amyloid and FDG-PET in clinical practice: differences between secondary and tertiary care memory units. J Alzheimer's Dis. (2018) 63:1025–33. 10.3233/JAD-170985 29710706

[18] Sánchez-Juan P Ghosh PM Hagen J Gesierich B Henry M Grinberg LT. Practical utility of amyloid and FDG-PET in an academic dementia center. Neurology. (2014) 82:230–8. 10.1212/WNL.0000000000000032 24353340 PMC3902757

[19] Patricio CM Gabriela C Julieta RM Marcos FS Federico N Griselda R. Concordance between 11C-PIB-PET and clinical diagnosis in a memory clinic. Am J Alzheimers Dis Other Demen. (2015) 30:599–606. 10.1177/1533317515576387 25817631 PMC10852556

[20] Coutinho AM Busatto GF de Gobbi Porto FH de Paula Faria D Ono CR Garcez AT. Brain PET amyloid and neurodegeneration biomarkers in the context of the 2018. NIA-AA research framework: an individual approach exploring clinical-biomarker mismatches and sociodemographic parameters. Eur J Nucl Med Mol Imaging. (2020) 47:2666–80. 10.1007/s00259-020-04714-0 32055966

[21] Apostolova LG Haider JM Goukasian N Rabinovici GD Chételat G Ringman JM. Critical review of the appropriate use criteria for amyloid imaging: effect on diagnosis and patient care. Alzheimer's Dement Diagn Assess Dis Monit. (2016) 5:15–22. 10.1016/j.dadm.2016.12.001 28054024 PMC5198877

[22] Bensaïdane MR Beauregard JM Poulin S Buteau FA Guimond J Bergeron D. Clinical utility of amyloid PET imaging in the differential diagnosis of atypical dementias and its impact on caregivers. J Alzheimer's Dis. (2016) 52:1251–62. 10.3233/JAD-151180 27104896

[23] Frederiksen KS Hasselbalch SG Hejl A-M Law I Højgaard L Waldemar G. Added diagnostic value of 11C-PiB-PET in memory clinic patients with uncertain diagnosis. Dement Geriatr Cogn Dis Extra. (2012) 2:610–21. 10.1159/000345783 23341826 PMC3551383

[24] Grundman M Pontecorvo MJ Salloway SP Doraiswamy PM Fleisher AS Sadowsky CH. Potential impact of amyloid imaging on diagnosis and intended management in patients with progressive cognitive decline. Alzheimer Dis Assoc Disord. (2013) 27:4–15. 10.1097/WAD.0b013e318279d02a 23203162

[25] Grundman M Johnson KA Lu M Siderowf A Dellagnello G Arora AK. Effect of amyloid imaging on the diagnosis and management of patients with cognitive decline: impact of appropriate use criteria. Dement Geriatr Cogn Disord. (2016) 41:80–92. 10.1159/000441139 26745445 PMC6625826

[26] Ossenkoppele R Prins ND Pijnenburg YAL Lemstra AW Van Der Flier WM Adriaanse SF. Impact of molecular imaging on the diagnostic process in a memory clinic. Alzheimer's Dement. (2013) 9:414–21. 10.1016/j.jalz.2012.07.003 23164552

[27] Schipke CG Peters O Heuser I Grimmer T Sabbagh MN Sabri O. Impact of beta-amyloid-specific florbetaben pet imaging on confidence in early diagnosis of Alzheimer's disease. Dement Geriatr Cogn Disord. (2012) 33:416–22. 10.1159/000339367 22814208

[28] Zannas AS Doraiswamy PM Shpanskaya KS Murphy KR Petrella JR Burke JR. Impact of 18F-florbetapir PET imaging of β-amyloid neuritic plaque density on clinical decision-making. Neurocase. (2014) 20:466–73. 10.1080/13554794.2013.791867 23672654

[29] Zwan MD Bouwman FH Konijnenberg E Van Der Flier WM Lammertsma AA Verhey FRJ. Diagnostic impact of [18F]flutemetamol PET in early-onset dementia. Alzheimer's Res Ther. (2017) 9:2. 10.1186/s13195-016-0228-4 28093088 PMC5240413

[30] Boccardi M Altomare D Ferrari C Festari C Guerra UP Paghera B. Assessment of the incremental diagnostic value of florbetapir F 18 imaging in patients with cognitive impairment: the incremental diagnostic value of amyloid PET with [18F]-florbetapir (INDIA-FBP) study. JAMA Neurol. (2016) 73:1417–24. 10.1001/jamaneurol.2016.3751 27802513

[31] Pontecorvo MJ Siderowf A Dubois B Doraiswamy PM Frisoni GB Grundman M. Effectiveness of florbetapir PET imaging in changing patient management. Dement Geriatr Cogn Disord. (2017) 44:129–43. 10.1159/000478007 28787712 PMC5806476

[32] Nordberg A Carter SF Rinne J Drzezga A Brooks DJ Vandenberghe R. A European multicentre PET study of fibrillar amyloid in Alzheimer's disease. Eur J Nucl Med Mol Imaging. (2013) 40:104–14. 10.1007/s00259-012-2237-2 22961445 PMC3510420

[33] Mallik A Drzezga A Minoshima S. Clinical amyloid imaging. Semin Nuclear Med. (2017) 47:31–43. 10.1053/j.semnuclmed.2016.09.005 27987555

[34] Youn JH Siksou M Mackin RS Choi JS Chey J Lee JY. Differentiating illiteracy from Alzheimer's disease by using neuropsychological assessments. Int Psychogeriatr. (2011) 23:1560–8. 10.1017/S1041610211001347 21777502

[35] Caramelli P Poissant A Gauthier S Bellavance A Gauvreau D Lecours AR. Educational level and neuropsychological heterogeneity in dementia of the Alzheimer type. Alzheimer Dis Assoc Disord. (1997) 11:9–15. 10.1097/00002093-199703000-00003 9071439

[36] Kim H Chey J. Effects of education, literacy, and dementia on the clock drawing test performance. J Int Neuropsychol Soc. (2010) 16:1138–46. 10.1017/S1355617710000731 20961480

[37] Fichman HC Fernandes CS Nitrini R Lourenço RA Paradela EM de P Carthery-Goulart MT. Age and educational level effects on the performance of normal elderly on category verbal fluency tasks. Dement Neuropsychol. (2009) 3:49–54. 10.1590/S1980-57642009DN30100010 29213610 PMC5619032

[38] Kosmidis MH. Challenges in the neuropsychological assessment of illiterate older adults. Language Cogn Neurosci. (2018) 33:373–86. 10.1080/23273798.2017.1379605

[39] Méndez PC Calandri I Nahas F Russo MJ Demey I Martín ME. Argentina-Alzheimer's disease neuroimaging initiative (Arg-ADNI): neuropsychological evolution profile after one-year follow up. Arq Neuropsiquiatr. (2018) 76:231–40. 10.1590/0004-282x20180025 29742242

[40] Allegri RF Chrem Méndez P Calandri I Cohen G Martín ME Russo MJ. Prognostic value of ATN Alzheimer biomarkers: 60-month follow-up results from the argentine Alzheimer's disease neuroimaging initiative. Alzheimer's Dement Diagn Assess Dis Monit. (2020) 12:e12026. 10.1002/dad2.12026 32490138 PMC7243942

[41] Russo MJ Cohen G Mendez PC Campos J Nahas FE Surace EI. Predicting episodic memory performance using different biomarkers: Results from Argentina-Alzheimer's disease neuroimaging initiative. Neuropsychiatr Dis Treat. (2016) 12:2199–206. 10.2147/NDT.S107051 27695331 PMC5028172

[42] Sanchez JS Hanseeuw BJ Lopera F Sperling RA Baena A Bocanegra Y. Longitudinal amyloid and tau accumulation in autosomal dominant Alzheimer's disease: findings from the Colombia-Boston (COLBOS) biomarker study. Alzheimer's Res Ther. (2021) 13:27. 10.1186/s13195-020-00765-5 33451357 PMC7811244

[43] Busatto GF de Gobbi Porto FH Faria D de P Squarzoni P Coutinho AM Garcez AT. *In vivo* imaging evidence of poor cognitive resilience to Alzheimer's disease pathology in subjects with very low cognitive reserve from a low-middle income environment. Alzheimer's Dement Diagn Assess Dis Monit. (2020) 12:e12122. 10.1002/dad2.12122 33426265 PMC7780143

[44] Parmera JB Coutinho AM Aranha MR Studart-Neto A de Godoi Carneiro C de Almeida IJ. FDG-PET patterns predict amyloid deposition and clinical profile in corticobasal syndrome. Mov Disord. (2020) 36:651–61. 10.1002/mds.28373 33206389

[45] Arbizu J Luquin MR Abella J de la Fuente-Fernández R Fernandez-Torrón R García-Solís D. Neuroimagen funcional en el diagnóstico de pacientes con síndrome parkinsoniano: actualización y recomendaciones para el uso clínico. Rev Esp Med Nucl Imagen Mol. (2015) 34:215–26. 10.1016/j.remn.2014.02.001 24731551

[46] Arbizu J García-Ribas G Carrió I Garrastachu P Martínez-Lage P Molinuevo JL. Recomendaciones para la utilización de biomarcadores de imagen PET en el proceso diagnóstico de las enfermedades neurodegenerativas que cursan con demencia: documento de consenso SEMNIM y SEN. Rev Esp Med Nucl Imagen Mol. (2015) 34:303–13. 10.1016/j.remn.2015.03.002 26099942

[47] Hill CV. Sankofa-highlighting legacy in the pursuit of equity for dementia science. JAMA Neurol. (2020) 78:271–27. 10.1001/jamaneurol.2020.4481 33252649

